# The Impact of a Six-Year Existing Screening Programme Using the Faecal Immunochemical Test in Flanders (Belgium) on Colorectal Cancer Incidence, Mortality and Survival: A Population-Based Study

**DOI:** 10.3390/ijerph20021654

**Published:** 2023-01-16

**Authors:** Thuy Ngan Tran, Sarah Hoeck, Harlinde De Schutter, Sharon Janssens, Marc Peeters, Guido Van Hal

**Affiliations:** 1 Centre for Cancer Detection, 8000 Bruges, Belgium; 2 Family Medicine and Population Health (FAMPOP), Faculty of Medicine and Health Sciences, University of Antwerp, 2610 Antwerp, Belgium; 3 Research Department, Belgian Cancer Registry, 1210 Brussels, Belgium; 4 Department of Oncology, Antwerp University Hospital, 2650 Edegem, Belgium; 5 Integrated Personalized & Precision Oncology Network (IPPON), University of Antwerp, 2610 Antwerp, Belgium

**Keywords:** colorectal cancer, cancer screening, incidence, mortality, impact, screen-detected cancer, interval cancer, survival

## Abstract

The faecal immunochemical test (FIT) has been increasingly used for organised colorectal cancer (CRC) screening. We assessed the impact of a six-year existing FIT screening programme in Flanders (Belgium) on CRC incidence, mortality and survival. The Flemish CRC screening programme started in 2013, targeting individuals aged 50–74 years. Joinpoint regression was used to investigate trends of age-standardised CRC incidence and mortality among individuals aged 50–79 years (2004–2019). Their 5-year relative survival was calculated using the Ederer II method. We found that FIT screening significantly reduced CRC incidence, especially that of advanced-stage CRCs (69.8/100,000 in 2012 vs. 51.1/100,000 in 2019), with a greater impact in men. Mortality started to decline in men two years after organised screening implementation (annual reduction of 9.3% after 2015 vs. 2.2% before 2015). The 5-year relative survival was significantly higher in screen-detected (93.8%) and lower in FIT non-participant CRCs (61.9%) vs. FIT interval cancers and CRCs in never-invited cases (67.6% and 66.7%, respectively). Organised FIT screening in Flanders clearly reduced CRC incidence (especially advanced-stage) and mortality (in men, but not yet in women). Survival is significantly better in screen-detected cases vs. CRCs in unscreened people. Our findings support the implementation of FIT organised screening and the continued effort to increase uptake.

## 1. Introduction

Worldwide, colorectal cancer (CRC) represents a considerable portion of the overall cancer burden, accounting for one in every ten cancer cases and deaths [1]. In Flanders, among the most common types of cancer, CRC ranks second in women after breast cancer and third in men after prostate and lung cancer. Concerning CRC alone, the CRC incidence is higher in men than in women, with age-standardised (world standard population) CRC incidence rates of 31.9/100,000 and 22.8/100,000 person-years, respectively, for men and women in 2020 [2].

Most CRCs develop from benign polyps over a long natural history of at least 10 years [3], and screening techniques are available for detecting and treating the disease at early premalignant stages, making CRC one of the most preventable cancers [4,5]. The European Guidelines recommend faecal occult blood test (FOBT) as the primary CRC screening tool [6]. The guaiac FOBT was reported to reduce CRC-related mortality by 15.0–33.0% [7,8]. More recently, the faecal immunochemical test (FIT) has been shown to be superior to the guaiac FOBT in terms of sensitivity, user-friendly sampling design and quantitative result [9].

Although several studies have demonstrated the effect of FIT screening on reducing CRC incidence and mortality, few of them used standardised parameter estimates [10,11], which restricts the comparison with other studies. Additionally, the magnitude of screening impact varies due to differences in screening uptake, background rate, length of follow-up and FIT cut-off. CRC mortality declined by 8.8% after 7 years of implementing biennial FIT screening (cut-off 20 µg Hb/g) in Spain [10] and a reduction of 52% was observed after 16 years of annual FIT screening (cut-off 20 µg Hb/g) in northern California (US) [12], while no significant reduction was observed after 6 years of biennial FIT screening (cut-off 47 µg Hb/g) in the Netherlands [11].

FIT screening programmes worldwide (in pilot phase or started recently) are currently using different screening strategies, with FIT cut-offs ranging from 15 to 80 µg Hb/g and screening intervals of one or two years, depending on their desired diagnostic value and colonoscopy capacity [13]. A number of countries and regions where no CRC screening programmes are in place yet are planning to implement one in the near future [14,15].

High-quality and generalisable data on the effectiveness of specific strategies for CRC screening are crucial to improve the existing screening programmes or to provide evidence for the initiation of a new one. Such information also enables the target population to make an informed decision about their screening participation. In this study, we investigated the impact of a six-year existing FIT organised screening programme in Flanders on age-standardised CRC incidence, mortality and relative survival.

## 2. Materials and Methods

### 2.1. The Flemish Organised CRC Screening Programme

The Flemish CRC screening programme, offering a free FIT (by mail) every two years, started in October 2013 with a stepwise implementation by age, resulting in all target ages of 50–74 years included in 2020 (2013: 66–74 years, only even ages; 2014: 56–74 years, only even ages; 2015–2016: 56–74 years; 2017: 55–74 years; 2018: 53–74 years; 2019: 51–74 years; 2020: 50–74 years). Exclusion criteria include a CRC diagnosis in the past 10 years, performance of a stool test in the past 2 years, a virtual colonoscopy in the past 4 years, a complete colonoscopy in the past 10 years or a colectomy. An FIT positivity cut-off of 15 µg Hb/g was used. Individuals with a FIT+ result are recommended to undergo a follow-up colonoscopy. In 2019, the response rate of the programme was 51.5%; the FIT sensitivity, positive predictive value and detection rate for invasive CRCs were 72.4%, 3.3% and 0.16%, respectively [16].

### 2.2. Study Design, Outcomes and Study Populations

Our study is a retrospective, observational, population-based study.

Firstly, we investigated trends of CRC incidence during 2004–2019 and mortality during 2004–2018 in the population aged 50–79 years. In addition to ages of 50–74 years (target screening ages), we also included ages of 75–79 years to capture the long-term effect of screening after people reach the upper age limit for screening.

Secondly, we assessed 5-year relative survival among individuals diagnosed with CRC at ages of 50–74 years during 2004–2019. Study subjects were censored at the date of death, end of the study period (15 July 2021) or date of the last follow-up when they were known to be alive. Five screening status subgroups were defined, including screen-detected CRC, FIT-interval cancer, post-colonoscopy CRC after a FIT+, CRC in FIT non-participants and never-invited (definitions in Table 1).

Our goal was to assess the impact of screening on the relative survival of individuals that adhered to the programme’s recommendations. Thus, we left out cases that were excluded from CRC screening invitation and those that had a deviated follow-up after a FIT+ result taken inside the organised screening programme, including another follow-up rather than a colonoscopy, a combination of different follow-up techniques or no follow-up at all. Since survival by stage was investigated in this study, cases that were suspected of having undergone pre-operative treatment (neo-adjuvant treatment)—presenting with a higher clinical vs. pathological stage—were also excluded.

### 2.3. Data Sources

Data on CRC incidence and tumour and patient characteristics were retrieved from the Belgian Cancer Registry (BCR) [17]. Tumour stage was determined using the applicable TNM edition at the time of diagnosis and was classified as early-stage (stages I and II) or advanced-stage (stages III and IV) [18,19,20]. Pathological staging was prioritised over clinical staging, except in the presence of clinical distant metastases, which were always considered stage IV. In the case of multiple lesions, the first primary invasive tumour was retained [12]. Demographic population data including life tables were retrieved from Statistics Belgium—Statbel (publicly available data) [21].

The following data were linked and transferred to the BCR for research purposes following authorisation (reference number 13/091) from the Committee for the Protection of Privacy, which is now the Information Security Committee [22,23]: data on individuals’ vital status were obtained from the data linkage between the BCR and the Belgian Crossroads Bank for Social Security (CBSS) based on social security number [24]. Cause of death was derived from the death certificates, collected by the regional authority (‘Agentschap Zorg en Gezondheid’ for Flanders). Data on FIT screening history (screening invitation, screening participation and FIT result) were extracted from the database of the Flemish Centre for Cancer Detection (CCD). Information on follow-up colonoscopy was based on reimbursement data from the Intermutualistic Agency (IMA-AIM) that are collected from seven health insurance companies in Belgium. These data are complete for 99% of cases due to the compulsory health insurance in Belgium.

### 2.4. Statistical Analysis

#### 2.4.1. Sample Size

In total, 55,688 invasive CRCs during 2004–2019 and 14,146 CRC-related deaths during 2004–2018 in people aged 50–79 years were included in the analysis of CRC incidence and mortality, and 35,796 CRCs in people aged 50–74 years during 2004–2019 were included in the analysis of relative survival.

#### 2.4.2. Missing Data

Throughout 2004–2019, the staging information of about 6.6% of CRCs was registered with an unspecified code or left blank by data providers. These CRCs were included in the “unknown” stage category in our analyses.

#### 2.4.3. Main Analysis

Age-specific and truncated age-standardised (world standard population) CRC incidence and mortality rates were calculated as the number of new CRC cases and CRC-related deaths, respectively, per 100,000 person-years (py) for each year during the study period.

Trends of CRC incidence and mortality were investigated using Joinpoint regression analyses. Changes in the evolution of the rates, indicated by joinpoints (where the slope of the regression function changes), were identified and annual percentage change (APC) was calculated.

Relative survival was calculated as the ratio of the observed and expected survival for a comparable group of the general population matched by age, sex and calendar year. The expected survival was calculated using the Ederer II method and the Flemish life tables [25,26]. The log-rank test was performed to compare survival rates among the screening status subgroups. 

We used the Joinpoint regression Software (version 4.9.0.0; US National Cancer Institute) and RStudio Software (version 1.3.1056; RStudio, PBC, Boston, MA, USA) for data analyses. A *p*-value of < 0.05 (two-sided) was considered statistically significant.

### 2.5. Privacy and Ethics

The secondary use and linkage of the databases involved was approved on 17 September 2013 (updated on 20 March 2018), with reference number 13/091, by the Committee for the Protection of Privacy, which is now the Information Security Committee [22,23]. Approval from an ethical committee was not necessary given the fact that this retrospective study does not fall under the Belgian legislation for ethical committee approval (Law of 7 May 2004 regarding experiments on human persons (art. 3, Section 2)). Participants in the Flemish CRC screening programme fill in a written informed consent agreeing that personal information can be used for scientific research and for the evaluation of the programme. Our reporting adheres to the STROBE guidelines for observational studies (Table S1) [27].

## 3. Results

**Figure 1:**
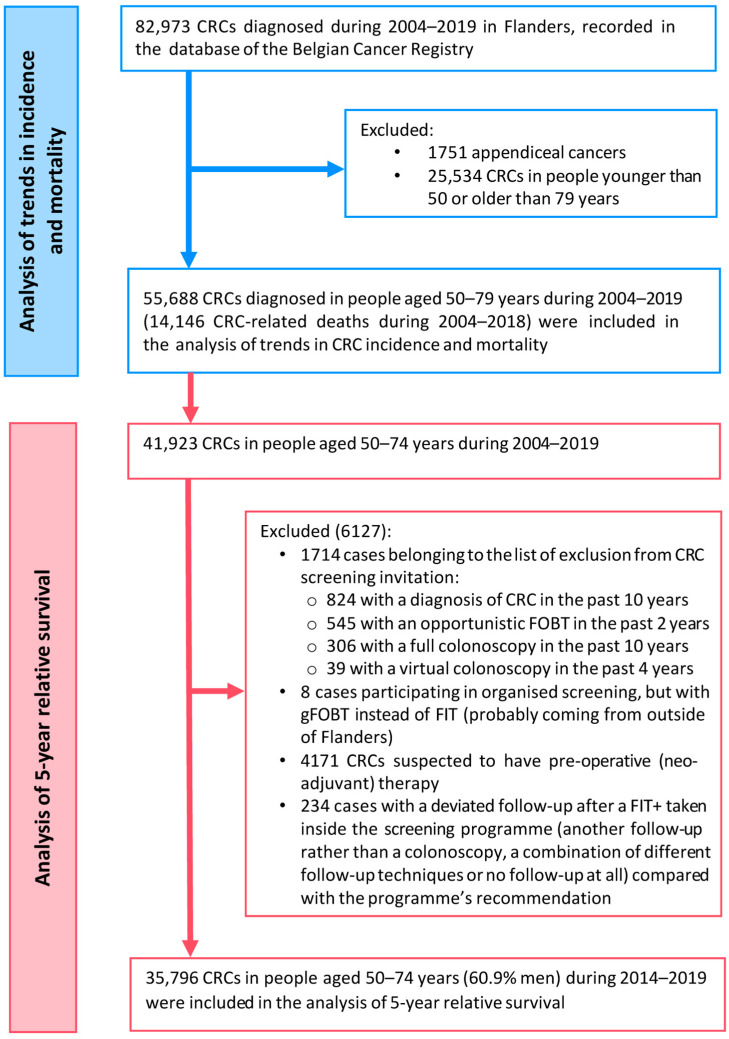
Flowchart of inclusion of study subjects in the study.

In total, 55,688 CRC cases during 2004–2019 and 14,146 CRC-related deaths during 2004–2018 in people aged 50–79 years were included in the analyses of CRC incidence and mortality trends. A subgroup of 35,796 CRCs in people aged 50–74 years during 2004–2019 was included in the analysis of 5-year relative survival (Figure 1).

### 3.1. Trends of CRC Incidence and Mortality by Gender

**Figure 2:**
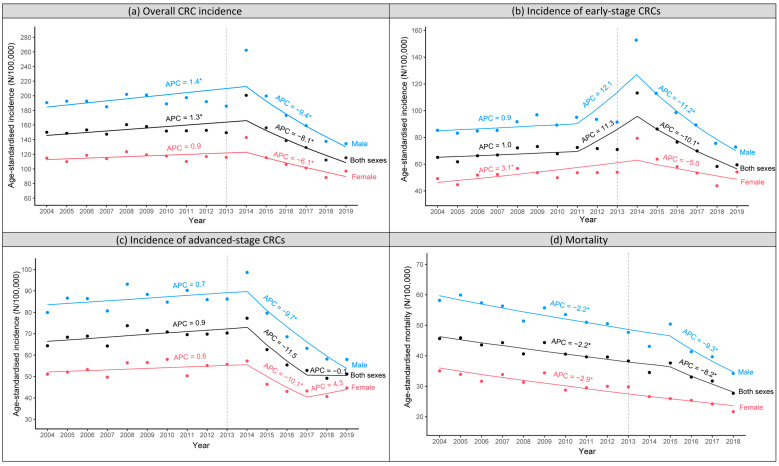
Trends of age-standardised CRC incidence and mortality, stratified by gender, in people aged 50–79 years in Flanders, Belgium, during 2004–2019 (incidence) and 2004–2018 (mortality). (**a**) Overall CRC incidence. (**b**) Incidence of early-stage CRCs. (**c**) Incidence of advanced-stage CRCs. (**d**) Mortality. The transparent dashed line presents the year when the organised colorectal cancer screening programme was initiated in Flanders. CRC, colorectal cancer; APC, annual percentage change; * statistically significant.

After the screening programme started in 2013, age-standardised CRC incidence initially increased and reached a peak in 2014 and then decreased drastically to a lower rate compared to before organised screening (134.5/100,000 py in 2019 vs. 191.9/100,000 py in 2012 for men; 96.9/100,000 py in 2019 vs. 116.9/100,000 py in 2012 for women). The impact was greater in men than in women. The annual percentage change (APC [95%CI]) for the periods before and after the peak in 2014 was 1.4% [0.6 to 2.3] and −9.4% [−11.4 to −7.3] for men and 0.9% [−0.2 to 2.0] and −6.1% [−9.1 to −3.1] for women, respectively (Figure 2a).

The increase in incidence after screening implementation was more pronounced for early-stage CRCs than advanced-stage CRCs. The early-stage CRC incidence rose sharply between 2013 and 2014 and then decreased steadily between 2014 and 2019 to a similar (for women) or slightly lower rate (for men). In contrast, the incidence of advanced-stage CRCs only increased slightly during 2013–2014 and then decreased drastically to a significantly lower rate compared to before the start of organised screening (58.0/100,000 py in 2019 vs. 85.9/100,000 py in 2012 for men; 44.7/100,000 py in 2019 vs. 55.1/100,000 py in 2012 for women). There was a slight increase in advanced-stage CRC incidence in women during 2017–2019 (APC 4.3%, not statistically significant) (Figure 2b,c).

Age-standardised CRC-related mortality was already decreasing gradually in both men and women before the implementation of organised screening. However, a sharper decline was observed in men starting from two years after the implementation of the screening programme (APC −9.3% [−15.2 to −3.0] after 2015 vs. −2.2% [−3.1 to −1.4] before 2015). No change in mortality trend during the study period was found in women (Figure 2d).

### 3.2. Trends of CRC Incidence and Mortality by Age Group

**Figure 3:**
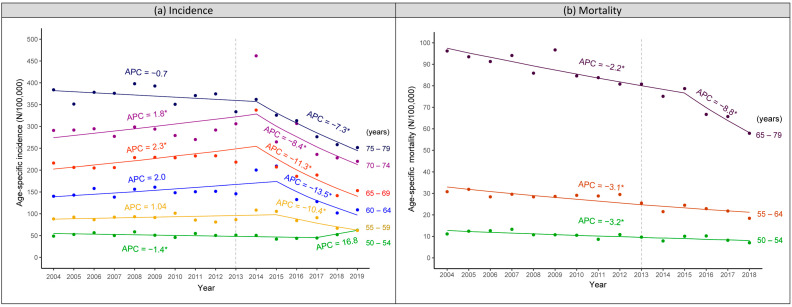
Trends of age-specific CRC incidence and mortality in people aged 50–79 years in Flanders, Belgium, during 2004–2019 (incidence) and 2004–2018 (mortality). (**a**) Incidence. (**b**) Mortality. The transparent dashed line presents the year when the organised colorectal cancer screening programme was initiated in Flanders. CRC, colorectal cancer; APC, annual percentage change; * statistically significant.

Figure 3a presents the trends of age-specific CRC incidence, which were completely in line with the stepwise introduction by age cohorts of the screening programme. Being included right from the start in 2013, the incidence in age groups of 65–69 and 70–74 years reached a peak in 2014. A similar peak was observed in 2015 for 55–59 and 60–64 years since these ages were only included since 2014. As the youngest group, 50–54 years, was only included since 2018, an increase in its incidence was observed during 2018–2019.

The impact of organised screening continued after screened individuals reached the upper age limit for screening. CRC incidence in the group of 75–79 years decreased substantially (APC −7.3% [−10.4 to −4.1]) during 2014–2019 to a significantly lower rate compared to before the start of the screening programme (251.6/100,000 py in 2019 vs. 374.6/100,000 py in 2012).

Although CRC-related mortality decreased gradually during the study period, no change in trend of mortality was identified when each 5-year age group was assessed separately (Figure S1). When the groups with a similar incidence trend (a peak reached in 2014 or 2015 or no peak observed yet) were combined, a change in the trend of mortality was captured for the group above 65 years, with a sharper decline from 2015 onwards (APC −8.8% [−14.7 to −2.5] after 2015 vs. −2.2% [−3.0 to −1.3] before 2015) (Figure 3b).

### 3.3. Relative Survival by Screening Status

In total, 4959 screen-detected CRCs, 905 FIT-interval cancers, 4555 CRCs in FIT non-participants and 25,353 CRCs in never-invited cases were included in the relative survival analyses. The results for the post-colonoscopy CRC subgroup are shown in our Supplementary Materials due to its small sample size (24 cases) (Table S2, Figures S2 and S3 and Supplementary Text).

Table 2 presents the study subjects’ characteristics. The majority in all subgroups were men (60.3–63.7%), except for the FIT-interval cancer group (sexes almost equally distributed). There was no major difference in the mean age among the subgroups (64.4–66.5 years). The mean time between FIT participation and CRC diagnosis was significantly longer among FIT-interval cancers vs. screen-detected CRCs (425.0 vs. 77.9 days). 

**Figure 4:**
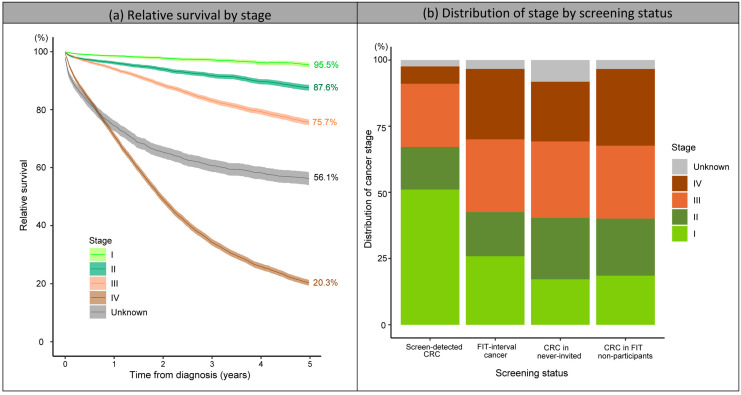
The 5-year relative survival by tumour stage and the distribution of tumour stage by screening status. (**a**) Relative survival by stage. (**b**) Distribution of stage by screening status. CRC, colorectal cancer; FIT, faecal immunochemical test.

**Figure 5:**
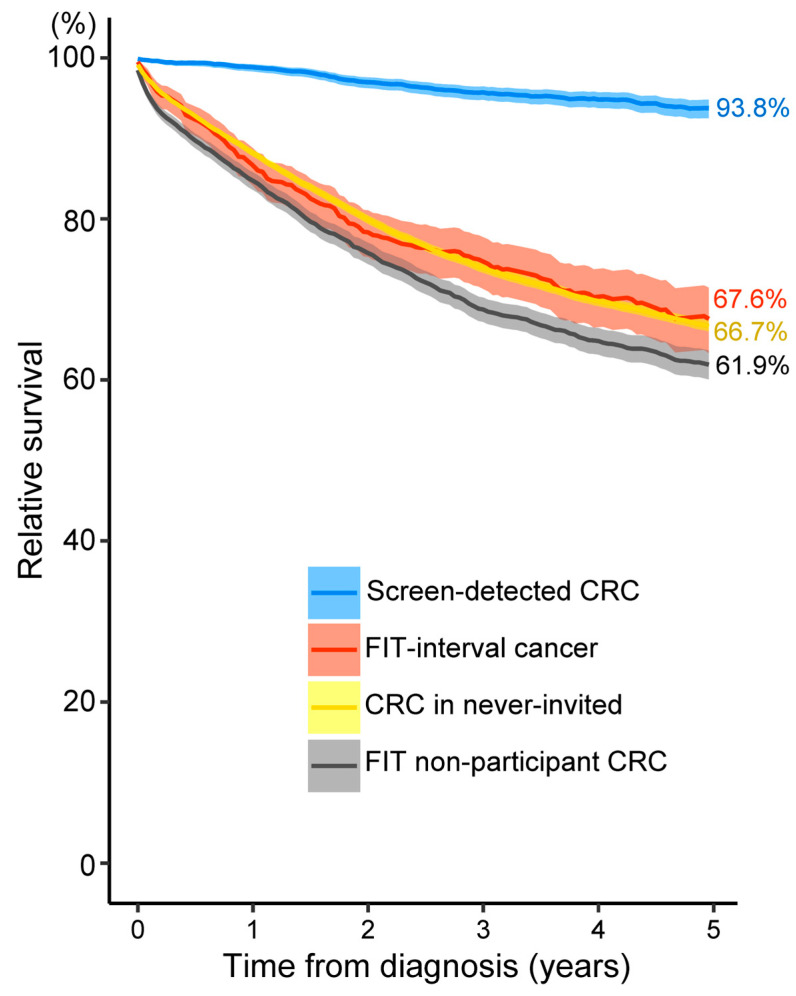
Five-year relative survival by screening status. CRC, colorectal cancer; FIT, faecal immunochemical test.

The 5-year relative survival decreased with increasing stage: 95.5% for stage I, 87.6% for stage II, 75.7% for stage III and only 20.3% for stage IV CRCs. The 5-year relative survival of unknown-stage CRCs was also low (56.1%) (Figure 4a). The proportion of early-stage CRCs (I or II) among the screen-detected CRCs was significantly higher than the other subgroups (67.1% vs. 40.1–42.6%) (Figure 4b and Table 2). As a result, the 5-year relative survival of screen-detected CRCs was significantly higher than the survival of the other subgroups (93.8% vs. 61.9–67.6%, *p*-values < 0.01) (Figure 5). Among CRCs in FIT non-participants, never-invited cases and FIT-interval cancers, CRCs in FIT non-participants had a significantly lower 5-year relative survival than the other two subgroups (61.9% vs. 66.7–67.6%, *p*-values < 0.01) (Figure 5) due to a higher proportion of advanced-stage CRCs (especially stage IV) compared to CRCs in never-invited cases and a lower proportion of early-stage CRCs (especially stage I) compared to FIT-interval cancers (Figure 4b and Table 2). 

## 4. Discussion

We investigated the impact of a six-year existing FIT screening programme in Flanders on CRC incidence, mortality and relative survival. The implementation of organised screening induced a sharp rise in early-stage CRC incidence, followed by a gradual decrease to a similar (women) or slightly lower rate (men). Conversely, advanced-stage CRC incidence only increased slightly but then decreased drastically to a significantly lower rate compared to before the implementation of organised screening. The impact of screening was more pronounced in men than in women and continued after individuals reached the upper age for screening. The effect of screening on mortality has already been observed in men and the group older than 65 years, but not yet in women and the younger groups. The 5-year relative survival was significantly higher in screen-detected CRCs and significantly lower in FIT non-participant CRCs compared to FIT-interval cancers and CRCs in never-invited cases, who shared a similar survival rate.

The observed trend of CRC incidence after the implementation of organised FIT screening in Flanders is similar to other countries where a high screening uptake (>50%) was rapidly achieved, such as the Netherlands, Slovenia and Denmark. A sharp increase in incidence (mostly early-stage CRCs) in the first 1–2 years after the initiation of the screening programme was noted in these countries, followed by a progressive decrease (both early- and advanced-stage CRCs) [11,15]. Organised screening thus resulted in an immediate increase in prevalent asymptomatic cases. These mainly comprised early-stage CRCs that, without organised screening, would take years to become symptomatic and be detected. In alignment with these observations, we observed a sharp peak in incidence right after the start of organised screening for early-stage CRCs but only a slight one for advanced-stage CRCs.

After the peak, however, advanced-stage CRC incidence decreased drastically to a significantly lower level than before the screening programme started. This decrease in advanced-stage CRC incidence resulted from a massive detection of early asymptomatic cases before they progressed into advanced stages. At the same time, screening enables the detection and removal of precursors, leading to a reduction in CRC incidence in the long term [15,28]. In Flanders, the overall CRC incidence decreased steadily from the peak in 2014 (200.6/100,000 py)—one year after the start of the programme—to a significantly lower level (115.2/100,000 py in 2019 vs. 152.7/100,000 py in 2012). A similar reduction in CRC incidence has been observed in Italy, Basque Country (Spain), northern California (US) and Taiwan [12,28,29,30].

The down-staging effect of CRC screening, i.e., shifting towards an earlier stage at diagnosis, has been well reported in the literature. In line with results from Slovenia, northern Italy and Basque Country (Spain) [31,32,33], almost 70% of CRCs detected within the Flemish screening programme, compared to only around 40% of CRCs in FIT-non-participants and never-invited cases, were in stages I and II. The Australian National Bowel Cancer Screening Program also reported 171% higher odds of being diagnosed at an earlier stage among screen-detected vs. screening non-participant CRCs [34].

The ultimate goal of CRC screening is to reduce CRC-related mortality through the down-staging effect (resulting in better treatment options and prognosis), together with a reduction in incidence in the long term [35,36]. Compared with CRC incidence, which is immediately influenced after screening implementation, the impact on CRC-related mortality is delayed and less pronounced [11]. In Flanders, CRC-related mortality was already decreasing steadily before the start of organised screening, probably due to improvements in treatment and patient care [37]. A steeper decline in mortality (indicating an additional impact of screening) was captured starting from two years after screening implementation, mainly in men (annual reduction of 9.3% after 2015 vs. 2.2% before 2015). Other FIT programmes have also reported various impacts of FIT screening on mortality according to levels of screening implementation, progress in incidence reduction, baseline mortality and length of follow-up [15]. Based on data from Italy, Spain, the US and Taiwan, FIT organised screening reduced CRC-related mortality by 9–52% after a follow-up duration of 6–16 years [10,12,28,29,36,38,39].

In line with previous studies [11,31,40], we observed a more pronounced impact of organised FIT screening on both CRC incidence and mortality in men than in women. There are two possible reasons: (1) CRC incidence and mortality are generally higher in men [13], and the effect is therefore expected to be greater in men; (2) the FIT is more sensitive in men [36,41]. A clear illustration shown in this study is the difference in the trend of advanced-stage CRC incidence during 2017–2019 between men and women (Figure 2c): the advanced-stage CRC incidence continued to decrease in men while it increased slightly in women. Such a slight increase in advanced-stage CRC incidence in women was observed because the decreasing pattern due to the detection (and treatment) of asymptomatic CRCs and precancerous lesions through active screening was cancelled out by the increasing pattern due to the entry of two new age cohorts each year in 2018 and 2019 in Flanders (leading to the detection of a large number of prevalent CRCs). In contrast, the decreasing pattern was still more predominant than the increasing pattern in men during this period. Likewise, a change in mortality trend (sharper decline) was already observed starting from two years after the start of the screening programme in men, but not yet in women.

Previous studies have suggested lowering the FIT cut-off or shortening the screening interval in women to narrow the gap in the test’s diagnostic performance between men and women [40,42,43,44,45]. However, our prior research has shown that in the context of the Flemish screening programme, where a low FIT cut-off of 15 µg Hg/g was already used for both sexes, lowering the FIT cut-off from 15 to 10 µg Hg/g or shortening the screening interval from two years to one year would only have a minimal impact on reducing FIT-interval cancers [41]. To address the reduced FIT sensitivity in women, new screening techniques may be required to replace or supplement the FIT for CRC screening in women [42,46,47].

Our findings aligned completely with the stepwise extension by age of the Flemish screening programme. The age cohorts included earlier experienced the effect of screening sooner. Specifically, those above 65 years old who entered the target population from the start in 2013 had a peak in incidence in 2014, while the 50–54 years group, included since 2017, had an increase in incidence during 2018–2019. The impact on mortality was already observed in those aged above 65 years but not yet in the younger population, for the following possible reasons: (1) CRC mortality is higher in the older population, and the impact is therefore more visible; (2) since ages above 65 years were included right from the start, these people could benefit from screening earlier with more screening rounds [29]; (3) since CRC takes years to develop and people with even advanced CRC still have a certain survival, time is required to observe an impact of screening on mortality. Therefore, the effect of screening on mortality is more apparent in older people who participated in screening in their earlier age. Notably, our results demonstrated that the screening effect continues after people reach the upper age limit for screening (i.e., 74 years). After six years of screening implementation, we have already observed a significant reduction in CRC incidence in the group aged 75–79 years.

In addition to incidence and mortality, we also investigated the impact of FIT screening on relative survival by screening status. In line with previous findings [30,31,32,33,48,49,50,51], we found that the 5-year relative survival was significantly higher among screen-detected CRCs (93.8%) and significantly lower among non-participant CRCs (61.9%) compared to FIT-interval cancers and CRCs in never-invited cases (67.6% and 66.7%, respectively). This finding strongly confirms the benefit of FIT screening on CRC-related survival and the importance of optimizing screening uptake in the target screening population. Note that, although considered undesirable events, the 5-year relative survival of FIT-interval cancers did not differ significantly from that of CRCs in never-invited cases (CRCs diagnosed when organised screening was not yet available).

The combined approach of evaluating the impact of FIT screening on both survival and mortality is an important strength of the current study. This combined approach minimised the influence of lead time bias on our interpretations of screening effects [52]. Specifically, if the increased survival in screen-detected CRCs was merely due to lead time bias (screening only brought forward time of diagnosis without affecting the disease course), CRC-related mortality would not have decreased after the implementation of organised FIT screening. We found the opposite in this study.

Our findings also did not support the theory of length time bias. According to this theory, screening would detect more slowly progressing cancers, leading to an overestimation of survival time in screen-detected CRCs. If this occurred, one would normally expect FIT-interval cancers—those escaping FIT screening—to have worse survival than non-screening CRCs. However, our findings and those from previous studies have shown a similar or even better survival in FIT-interval cancers than in CRCs diagnosed without screening [33,34,53]. Note that this result might also be affected by the healthy user bias, i.e., that subjects who participated in screening are likely to be healthier than those who did not [54]. Future research taking into account subjects’ lifestyles and health-seeking behaviours as well as length time bias is needed to validate the findings from ours and previous studies which showed that survival in FIT interval cancers is better than or similar to that of CRCs diagnosed without screening [33,34,53,54].

Another strength of this study is the use of register-based data, which eliminated information and selection biases. Moreover, we used the relative survival parameter in which survival of the CRC population is compared with the matched (age, sex and calendar year) general population. Thus, age, sex and improvement of general treatment and care over time (proxied by calendar year) were sufficiently controlled for in our analysis. Improvement in CRC-specific treatment was, however, only partially adjusted for with the use of relative survival since the CRC population benefits from CRC-specific treatment advances to a greater extent compared with the general population. Other potential biases due to opportunistic screening, pre-operative treatment and specific reasons leading to exclusion from CRC screening were also considered in our methodologies.

We could not account for changes in lifestyle factors over time in our trend analyses. Nevertheless, we expected the magnitude of such an influence on our results to be small due to two reasons: (1) there is no evidence of a substantial change in the adoption of low-CRC-risk behaviours in Flanders during the study period [15]; (2) a general change in lifestyle would induce similar trends in early- and advanced-stage CRC incidence. However, we observed totally different patterns in incidence for early- and advanced-stage CRCs after the implementation of the screening programme, for which screening is apparently a more plausible explanation.

## 5. Conclusions

Our data showed a clear impact of FIT organised screening on improving CRC survival and reducing incidence and mortality, with a more pronounced effect in men than in women. The impact of screening continued after people reached the upper target age for screening (i.e., older than 74 years). Our findings support the timely implementation of organised FIT screening programmes where they are not yet in place and the improvement of the existing ones. To maximise the impact of screening, increasing screening uptake is crucial.

## Figures and Tables

**Table 1:** Definitions of screening status subgroups.

Subgroup	Definition
Screen-detected CRC	CRC diagnosed after a FIT+ result, within six months after the first follow-up colonoscopy and before the next recommended FIT invitation (24 months).
FIT-interval cancer	CRC diagnosed after a negative FIT result and before the next recommended FIT invitation (24 months).
Post-colonoscopy CRC after a FIT+	CRC diagnosed after a FIT+ result but later than six months after the first follow-up colonoscopy and before the next recommended colonoscopy examination (10 years, 4 years and 2 years for a complete, virtual and incomplete colonoscopy, respectively).
CRC in FIT non-participant	CRC diagnosed but no FIT participation recorded after screening invitation.
CRC in never-invited *	CRC occurred before the start of the screening programme. CRC occurred after the start of the screening programme but in individuals whose ages were not yet included in the target screening ages at the time (e.g., age 50 during 2013–2019).

CRC, colorectal cancer; FIT, faecal immunochemical test. * CRC cases that were diagnosed during an individual’s period of exclusion from CRC screening (a CRC diagnosis ≤ 10 years, an opportunistic FOBT ≤ 2 years, a full colonoscopy ≤ 10 years or a virtual colonoscopy ≤ 4 years) were excluded from our analysis of relative survival.

**Table 2:** Characteristics of study subjects included in the analysis of 5-year relative survival: people aged 50–74 years at time of CRC diagnosis during 2004–2019 in Flanders, Belgium (grouped by CRC screening status).

	Screen-Detected CRC (N = 4959)	FIT-Interval Cancer (N = 905)	CRC in Never-Invited (N = 25,353)	FIT Non-Participant CRC (N = 4555)
Men	3157 (63.7%)	468 (51.7%)	15,298 (60.3%)	2854 (62.7%)
Mean age (years) ± SD	65.7 ± 5.8	66.5 ± 5.4	64.4 ± 6.9	66.2 ± 5.6
Stage				
• I	2532 (51.0%)	234 (25.9%)	4352 (17.2%)	845 (18.6%)
• II	799 (16.1%)	151 (16.7%)	5880 (23.2%)	980 (21.5%)
• III	1185 (23.9%)	249 (27.5%)	7325 (28.9%)	1257 (27.6%)
• IV	325 (6.6%)	241 (26.6%)	5723 (22.6%)	1321 (29.0%)
• Unknown	118 (2.4%)	30 (3.3%)	2073 (8.2%)	152 (3.3%)
Mean time between FIT and diagnosis (days)	77.9	425.0	-	-

CRC, colorectal cancer; FIT, faecal immunochemical test.

## Data Availability

The cancer cohort data used and analysed during the study are available from the corresponding author upon reasonable request. The pseudonymised data can be provided within the secured environment of the Belgian Cancer Registry after having been guaranteed that the applicable GDPR regulations are applied.

## Supplementary Materials

The following supporting information can be downloaded at: https://www.mdpi.com/article/10.3390/ijerph20021654/s1, Table S1: The STROBE research checklist for observational studies in epidemiology applied in the current study; Table S2: Characteristics of the study subjects in subgroups by screening status, with the post-colonoscopy CRC subgroup included (the last column); Supplementary Text: Relative survival of the post-colonoscopy colorectal cancer subgroup; Figure S1: Trends of age-specific CRC mortality in people aged 50–79 years in Flanders, Belgium, during 2004–2018 (by individual 5-year age group); Figure S2: Distribution of CRC stage by screening status, with the post-colonoscopy CRC subgroup included; Figure S3. Five-year relative survival by screening status, with the post-colonoscopy CRC subgroup included.
