# Supplementary material for: The Impact of a Six-Year Existing Screening Programme Using the Faecal Immunochemical Test in Flanders (Belgium) on Colorectal Cancer Incidence, Mortality and Survival: A Population-Based Study
Source: Int J Environ Res Public Health. 2023 Jan 16;20(2):1654. doi: 10.3390/ijerph20021654 (PMC9864341; doi:10.3390/ijerph20021654)
Supplement: Supplementary file 1 [file ijerph-20-01654-s001.zip › ijerph-2123013-supplementary.pdf]

Supplementary materials for

The impact of a six-year existing screening programme using the faecal immunochemical test in Flanders (Belgium) on colorectal cancer incidence, mortality and survival: a population-based study

Supplementary Materials: **Table S1.** The STROBE research checklist for observational studies in epidemiology applied in the current study. **Table S2.** Characteristics of the study subjects in subgroups by screening status, with the post-colonoscopy CRC subgroup included (the last column). **Supplementary text.** Relative survival of the post-colonoscopy colorectal cancer subgroup. **Figure S1.** Trends of age-specific CRC mortality in people aged 50-79 years in Flanders, Belgium during 2004-2018 (by individual 5-year age group). **Figure S2.** Distribution of CRC stage by screening status, with the post-colonoscopy CRC subgroup included. **Figure S3.** Five-year relative survival by screening status, with the post-colonoscopy CRC subgroup included.

Table S1 – The STROBE research checklist for observational studies in epidemiology applied in the current study.

	Item No	Recommendation	Page No.
**Title and abstract**	1	Title	1
		Provide in the abstract an informative and balanced summary of what was done and what was found	1
**Introduction**			
Background/rationale	2	Explain the scientific background and rationale for the investigation being reported	1-2
Objectives	3	State specific objectives, including any prespecified hypotheses	2
**Methods**			
Study design	4	Present key elements of study design early in the paper	2
Setting	5	Describe the setting, locations, and relevant dates, including periods of recruitment, exposure, follow-up, and data collection	2-4
Participants	6	Give the eligibility criteria, and the sources and methods of selection of participants. Describe methods of follow-up	2-3<br>Figure 1
		(*b*) For matched studies, give matching criteria and number of exposed and unexposed	Not applicable
Variables	7	Clearly define all outcomes, exposures, predictors, potential confounders, and effect modifiers.	2-4
Data sources/ measurement	8	For each variable of interest, give sources of data and details of methods of assessment (measurement). Describe comparability of assessment methods if there is more than one group	2-4
Bias	9	Describe any efforts to address potential sources of bias	3-4 (combined with 14&15 - Discussion)
Study size	10	Explain how the study size was arrived at	4-5
Quantitative variables	11	Explain how quantitative variables were handled in the analyses.	10 (Table 2)<br>Figure 3 shows how age was categorised
Statistical methods	12	Describe all statistical methods, including those used to control for confounding	4
		Describe any methods used to examine subgroups and interactions	4
		Explain how missing data were addressed	4
		If applicable, explain how loss to follow-up was addressed	2
		Describe any sensitivity analyses	Not applicable
**Results**			
Participants	13	Report numbers of individuals at each stage of study	4-5
		Give reasons for non-participation at each stage	Not applicable
		Consider use of a flow diagram	Figure 1
Descriptive data	14	Characteristics of study participants	10
		Number of participants with missing data	4
		Follow-up time	4 (combined with 2 – Methods)
Outcome data	15	Report numbers of outcome events or summary measures over time	4
Main results	16	Give unadjusted estimates and, if applicable, confounder-adjusted estimates and their precision (eg, 95% confidence interval).	5-12

		Make clear which confounders were adjusted for and why they were included	
		Report category boundaries when continuous variables were categorized	Not applicable
		If relevant, consider translating estimates of relative risk into absolute risk for a meaningful time period	Not applicable
Other analyses	17	Report other analyses done—eg analyses of subgroups and interactions, and sensitivity analyses	10-12
**Discussion**			
Key results	18	Summarise key results with reference to study objectives	12
Limitations	19	Discuss limitations of the study, taking into account sources of potential bias or imprecision. Discuss both direction and magnitude of any potential bias	14-15
Interpretation	20	Give a cautious overall interpretation of results considering objectives, limitations, multiplicity of analyses, results from similar studies, and other relevant evidence	12-15
Generalisability	21	Discuss the generalisability (external validity) of the study results	14-15
**Other information**			
Funding	22	Give the source of funding and the role of the funders for the present study and, if applicable, for the original study on which the present article is based	15

Table S2. Characteristics of the study subjects in subgroups by screening status, with the post-colonoscopy CRC subgroup included (the last column).

	**Screen-detected CRC (N=4,959)**	**FIT-interval cancer (N=905)**	**CRC in never-invited (N=25,353)**	**FIT non-participant CRC (N=4,555)**	**Post-colonoscopy CRC (N=24)**
Men	3,157 (63.7%)	468 (51.7%)	15,298 (60.3%)	2,854 (62.7%)	14 (58.3%)
Mean age (years) ± SD	65.7 ± 5.8	66.5 ± 5.4	64.4 ± 6.9	66.2 ± 5.6	68.5 ± 4.9
Mean time between FIT and diagnosis (days)	77.9	425.0	-	-	993.8
Stage					
I	2,532 (51.0%)	234 (25.9%)	4,352 (17.2%)	845 (18.6%)	3 (12.5%)
II	799 (16.1%)	151 (16.7%)	5,880 (23.2%)	980 (21.5%)	0 (0%)
III	1185 (23.9%)	249 (27.5%)	7,325 (28.9%)	1257 (27.6%)	12 (50%)
IV	325 (6.6%)	241 (26.6%)	5,723 (22.6%)	1321 (29.0%)	8 (33.3%)
Unknown	118 (2.4%)	30 (3.3%)	2,073 (8.2%)	152 (3.3%)	1 (4.2%)

CRC: Colorectal cancer; FIT, Faecal immunochemical test

Supplementary text:

Relative survival of the post-colonoscopy colorectal cancer subgroup

As introduced in the main manuscript, we present our results on individual relative survival of the "post-colonoscopy colorectal cancer (CRC) after an organised FIT+" subgroup in this Supplementary Materials due to the small sample of the subgroup (24 cases during 2013-2019). Similar to the screen-detected CRC, FIT non-participant and never-invited subgroups, the majority of the post-colonoscopy CRC subgroup were men (58.3%) and its mean age at diagnosis was slightly higher than the other subgroups (68.5 vs. 64.4-66.5 years, respectively). The mean time between FIT participation and CRC diagnosis among post-colonoscopy CRCs were 12.8 times and 2.3 times longer than screen-detected CRCs and FIT-interval cancers (Table S2). With regards to tumour stage distribution, 83.3% of CRCs in this subgroup were at an advanced stage III or IV while this proportion among screen-detected CRCs was only 30.5% and among FIT-interval cancers, CRCs in FIT non-participants and never-invited was 51.5-56.6% (Table S2 and Figure S2).

Among the subgroups by screening status investigated in this study, post-colonoscopy CRCs had the lowest 5-year relative survival of 50.9% (screen-detected CRCs: 93.8%, FIT-interval cancers: 67.6%, CRC in never-invited: 66.7%, CRCs in FIT non-participant CRCs: 61.9%) (Figure S3). When comparing with the 5-year relative survival between post-colonoscopy CRCs vs. the other subgroups, significance level was only reached in the comparison between post-colonoscopy CRCs and screen-detected CRCs (due to a large difference of >40%) but not in the comparisons with the other subgroups (due to the limited number of post-colonoscopy CRCs: 24 cases). To sufficiently study the survival of post-colonoscopy CRCs after FIT+, a longer study period (to provide an adequate sample size) or a different study methodology is required.

Figures S1 to S3

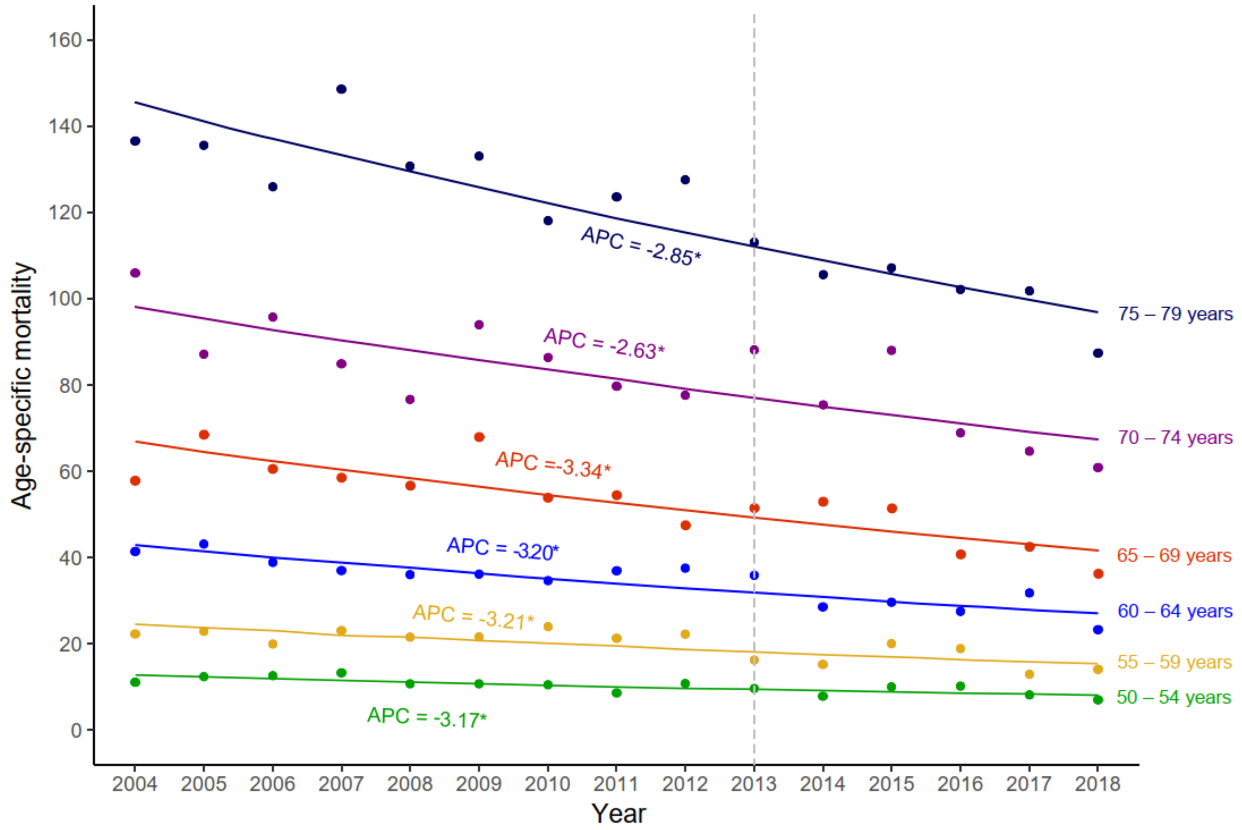

Figure S1. Trends of age-specific CRC mortality in people aged 50-79 years in Flanders, Belgium during 2004-2018 (by individual 5-year age group). The transparent dashed line presents the year when the organised colorectal cancer screening programme was initiated in Flanders. CRC, colorectal cancer; APC, annual percentage change; *statistically significant

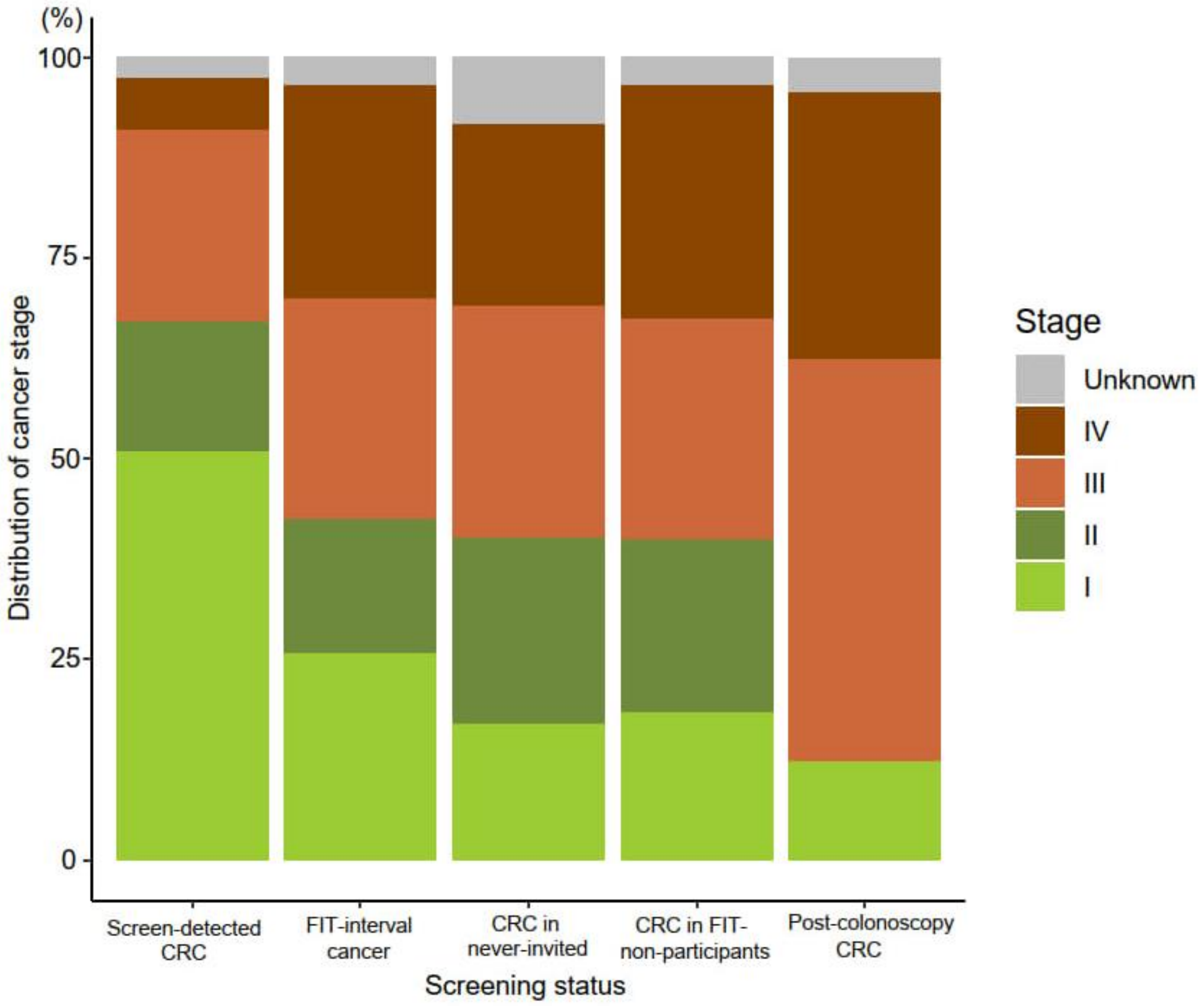

Figure S2. Distribution of CRC stage by screening status, with the post-colonoscopy CRC subgroup included. CRC, colorectal cancer, FIT, faecal immunochemical test

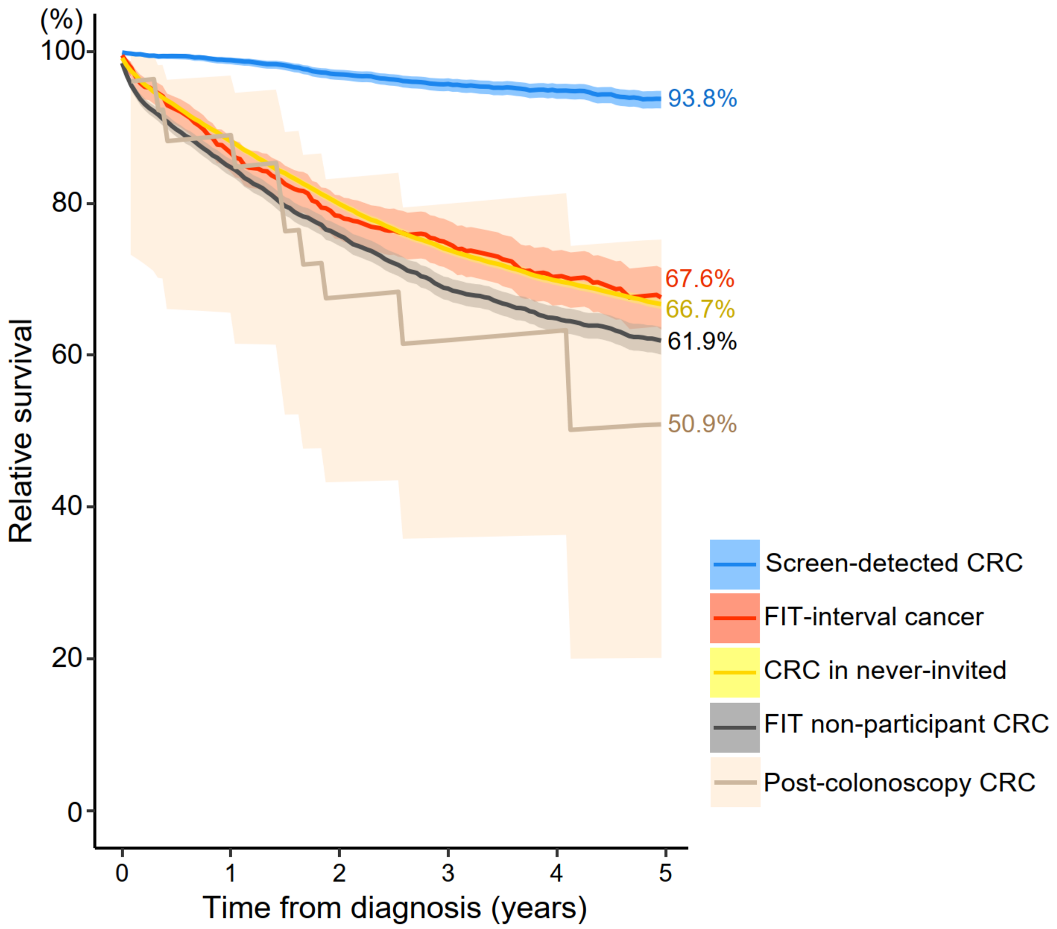

Figure S3. Five-year relative survival by screening status, with the post-colonoscopy CRC subgroup included. CRC, colorectal cancer, FIT, faecal immunochemical test.
